# Cancer Vaccines Designed Based the Nanoparticle and Tumor Cells for the Treatment of Tumors: A Perspective

**DOI:** 10.1049/2024/5593879

**Published:** 2024-02-24

**Authors:** Qing-Juan Wu, Wen-Liang Lv

**Affiliations:** Guang 'anmen Hospital, China Academy of Chinese Medical Sciences, Beijing, China

## Abstract

Cancer vaccines based on tumor cell components have shown promising results in animal and clinical studies. The vaccine system contains abundant tumor antigen components, which can activate the immune system by antigens. However, their efficacy has been limited by the inability of antigens delivery, which are the core components of vaccines, further fail to be presented and activation of effective cells. Nanotechnology offers a novel platform to enhance the immunogenicity of tumor-associated antigens and deliver them to antigen-presenting cells (APCs) more efficiently. In addition, nanotreatment of tumor cells derivate active ingredients could also help improve the effectiveness of cancer vaccines. In this review, we summarize recent advances in the development of cancer vaccines by the combination of nanotechnology and tumor-based ingredients, including liposomes, polymeric nanoparticles, metallic nanoparticles, virus-like particles and tumor cells membrane, tumor lysate, and specific tumor antigens. These nanovaccines have been designed to increase antigen uptake, prolong antigen presentation, and modulate immune responses through codelivery of immunostimulatory agents. We also further discuss challenges and opportunities in the clinical translation of these nanovaccines.

## 1. Introduction

Cancer immunotherapy has revolutionized cancer treatment by harnessing the immune system to recognize and attack cancer cells [1–3]. Among various immunotherapeutic approaches, cancer vaccines have attracted significant attention due to their potential to induce long-lasting and specific immune responses against tumor cells [4–7]. By comparison with surgery, it hardly produce large wounds, nor does it cause higher toxicity in normal tissues and drug tolerance like chemotherapy in the treatment of tumor. However, the clinical efficacy of cancer vaccines based on tumor cell components, such as peptides and proteins, is limited by their poor immunogenicity and inefficient delivery to antigen presentation cells (APCs). Although, many cancer vaccines were applied with the immune-stimulatory agents, such as the programmed cell death protein 1 (PD-1), CpG oligonucleotide, etc. The clinical response rates remain low, especially in the hypoimmunogenic tumors. As we all know, pancreatic cancer is a more difficult type for immunotherapy as a low immunogenic tumor. Therefore, there are few effective cancer vaccines that can actively promote the pancreatic cancer treatment. Meanwhile, limited by the specific tumor antigens, the development of cancer vaccines was extremely difficult.

The field of cancer vaccines has seen significant advancements in recent years, with numerous studies focusing on different aspects of vaccine development and application [8–12]. These advances have been facilitated by the exploration of various types of cancer vaccines, including those targeting viral antigens, neoantigens, and other tumor-specific antigens [13–16]. One approach in cancer vaccine research involves targeting shared antigens from viral infections linked to certain cancer types, such as Epstein–Barr virus and HPV. Studies have shown promising results with vaccines targeting viral antigens like Epstein–Barr nuclear antigen 1 and latent membrane proteins, as well as HPV-related antigens, demonstrating favorable antitumor efficacy and the potential to establish durable T cell memory in rapidly progressing HPV-positive tumors [17]. Another significant area of research is neoantigen cancer vaccines. Neoantigens, derived from nonsynonymous cell variants, are only expressed in tumor cells and can bypass thymus-negative selection, leading to robust neoantigen-specific T cell responses [14]. Advances in genomic and transcriptional profiling have made identifying putative neoantigens possible, although challenges remain in predicting which neoantigens can effectively induce neoantigen-specific T cell responses [18–20]. Cancer vaccines are being developed to stimulate antitumor immunity with tumor antigens delivered in various forms, such as cells, peptides, viruses, and nucleic acids [21–24]. These vaccines aim to overcome tumor heterogeneity and reverse the immunosuppressive microenvironment. Challenges in the field include single antigen targeting, weak immunogenicity, off-target effects, and impaired immune response [25–27]. Current research and reviews provide insights into the principle of action, components, classification, delivery methods, and progress of cancer vaccines, offering a broader perspective for future vaccine design.

**Figure 1:**
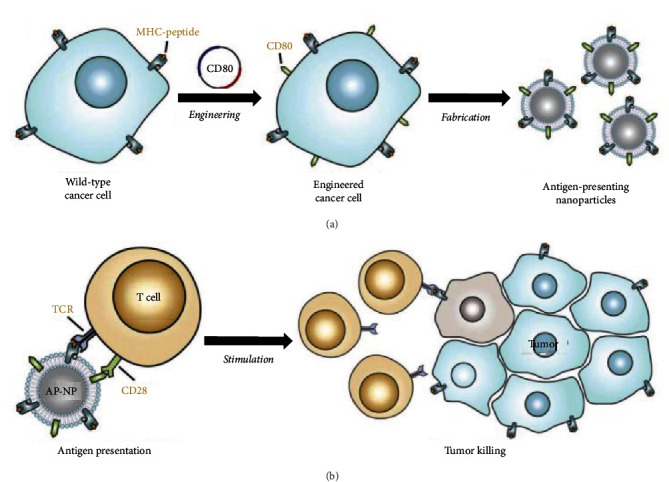
Engineered cell-membrane-coated nanoparticles for direct antigen presentation: (a) wild-type cancer cells are induced the generation of CD80 to present the antigens. Then, the membrane is collected for the encapsulation of polymeric nanoparticle cores and (b) the antigen-presenting nanoparticles (AP-NPs) can directly stimulate tumor antigen-specific T cells and further kill the same tumors [35].

Recent studies have shown that tumor antigens can be used for the design of cancer vaccines without the necessity to identify, parse, and reconstruct certain antigens [28–31]. A novel concept has been proposed that the whole antigen vaccine systems derived from tumor cells could be designed to conquer cancer treatments [32–34]. The tumor cells themselves are a huge antigen pool with countless tumor antigen epitopes, which can be used to activate antitumor immune response. Among them, more studies have focused on the application of tumor cell membrane as tumor antigens to stimulate APCs and further activate the antitumor immune response of T cells. In addition, the activation of T cells in immune response requires the involvement of costimulatory molecules. Some studies are based on this theoretical foundation, using genetic engineering to modify tumor cell membranes. By retaining antigen on the cell membrane and inducing the expression of costimulatory molecule CD80, T cells can be directly activated, thereby achieving antitumor immune response [35] (Figure 1). Of course, many studies combine tumor cell membranes that retain tumor antigens with other functional molecules to directly activate dendritic cells (DCs) for antigen presentation, leading to the activation of T cell immune response. These are two different approaches aimed at achieving the same goal [36–38]. A series of nanovaccines developed on this basis have been widely used in the treatment of different tumor cells and preclinical experiments.

**Figure 2:**
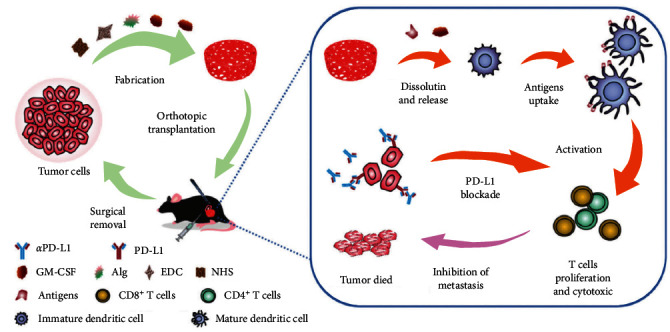
Schematic illustration of hydrogel derived from tumor cell lysate preparation and hydrogel-mediated cancer immunotherapy to prevent postoperative tumor recurrence and metastasis [39].

In addition to utilizing tumor cell membranes as a source of antigens, tumor cell lysate is also commonly used to activate antitumor immune response. Compared to extracting tumor cell membranes, its advantage lies in easy accessibility and fewer complex steps. For example, professor Deng prepared a cell lysate gel using homologous tumor cells to retain all antigens. By combining it with granulocyte–macrophage colony-stimulating factor (GM-CSF) that recruits DCs, DCs were recruited into the hydrogel, enabling them to uptake the tumor cell lysate. This completes antigen presentation and activates downstream T cell immune response, resulting in the suppression of tumor growth [39] (Figure 2).

Nanoparticles, such as liposomes, polymeric nanoparticles, and metal nanoparticles, have been widely explored for the development of cancer vaccines. These nanoscale particles offer unique advantages in delivering antigens and adjuvants to immune cells, enhancing vaccine efficacy. Liposomes are lipid-based nanoparticles that can encapsulate tumor antigens and facilitate their uptake by APCs. Polymeric nanoparticles provide a versatile platform for antigen loading and controlled release, allowing for sustained immune activation. Metal nanoparticles, such as gold nanoparticles, offer the potential for targeted delivery and enhanced immune responses through their unique physicochemical properties. Overall, the use of these diverse nanoparticles holds promise in the development of cancer vaccines, providing opportunities for improved immunotherapy strategies [40–42].

Several studies have investigated the use of various types of nanoparticles for the delivery of tumor cell components and adjuvants30−33. Lipid-based nanoparticles, such as liposomes and solid lipid nanoparticles (SLNs), have been widely used due to their biocompatibility and ability to encapsulate both hydrophilic and hydrophobic compounds. For example, a recent study used SLNs loaded with a synthetic long peptide (SLP) derived from the HPV16 E7 oncoprotein and the TLR7/8 agonist R848 to induce E7-specific CD8^+^ T cell responses in mice. The results showed that SLN-encapsulated SLP-R848 was more effective in inducing antitumor immune responses compared to the free SLP-R848, as evidenced by the increased production of IFN-*γ* and granzyme B by CD8^+^ T cells. Another type of nanoparticle that has shown promise in cancer vaccine development is polymeric nanoparticles. Polymeric nanoparticles are biodegradable and can be synthesized with a variety of sizes and surface modifications, making them ideal for targeted delivery to APCs34. A recent study used polymeric nanoparticles conjugated with MUC1 glycopeptides and a TLR9 agonist to induce MUC1-specific CD8^+^ T cell responses in mice. The results showed that the polymeric nanoparticles induced a robust immune response and provided significant protection against MUC1-expressing tumor cells. In addition to lipid-based and polymeric nanoparticles, inorganic nanoparticles have also been investigated for cancer vaccine development. For example, gold nanoparticles have been used to deliver tumor-associated antigens (TAAs) to DCs, resulting in increased activation and maturation of DCs and improved antitumor immune responses. Similarly, silica nanoparticles have been used to deliver TAAs and adjuvants to DCs, resulting in enhanced T cell activation and tumor suppression.

### 1.1. Liposomes

Liposomes are one of the most widely used nanocarriers for cancer vaccine delivery due to their biocompatibility and versatility in encapsulating various types of antigens and immunostimulatory agents [43–45]. For example, a liposomal vaccine composed of survivin peptide and monophosphoryl lipid A (MPLA) has been shown to elicit strong cytotoxic T lymphocyte (CTL) responses and inhibit tumor growth in a mouse model. In another study, a liposomal vaccine loaded with melanoma antigen peptides and toll-like receptor 7 (TLR7) agonist showed enhanced activation of DCs and antigen-specific T cells in vitro and in vivo. In addition, liposomes can be engineered to target specific cell types by conjugating ligands such as antibodies and peptides. For instance, a liposomal vaccine decorated with anti-CD205 antibody induced higher DC activation and antigen-specific T cell responses than untargeted liposomes.

### 1.2. Polymeric Nanoparticles

Polymeric nanoparticles have emerged as another promising platform for cancer vaccine delivery due to their tunable properties, biocompatibility, and biodegradability. One strategy to enhance the immunogenicity of polymeric nanoparticles is to incorporate immunostimulatory agents such as CpG oligonucleotides and interleukin-2 (IL-2) into the particle matrix. For example, a poly(lactic-co-glycolic acid; PLGA) nanoparticle vaccine containing ovalbumin peptide and CpG induced robust CTL responses and tumor inhibition in a mouse model. Another approach is to modify the surface of polymeric nanoparticles with functional groups such as amine and carboxyl groups to enhance antigen loading and targeting. A recent study reported that a hyaluronic acid-coated PLGA nanoparticle vaccine carrying NY-ESO-1 antigen and TLR7 agonist showed improved DC activation and antigen-specific T cell responses in vitro and in vivo.

### 1.3. Metallic Nanoparticles

Metallic nanoparticles, such as gold and silver nanoparticles, have unique optical and electronic properties that can be exploited for cancer vaccine development [46, 47]. For example, gold nanoparticles can absorb and scatter light, leading to photothermal effects and enhanced antigen uptake by APCs. A recent study demonstrated that a gold nanoparticle vaccine conjugated with HER2 peptide and TLR7 agonist induced strong CTL responses and tumor regression in a mouse model. Another application of metallic nanoparticles is to enhance antigen presentation by crosslinking peptide.

### 1.4. Vesicles Particles

Exosomes, as the most prominent type of vesicles derived from cells, have been extensively explored and applied in various therapeutic research studies for different diseases, including immunotherapy for tumors. Tumor cells are known to produce exosomes in high quantities, which possess homing abilities, carry tumor-specific antigens on their surface, and play important roles in cancer progression from the early stages to metastasis. Numerous studies have indicated that drug-loaded exosomes derived from tumors can effectively reduce the number of cancer cells and improve patient survival [48–50]. In addition, exosomes derived from various immune cells, such as macrophages and DCs, have been shown to possess the ability to inhibit tumor growth [50–52]. Therefore, drug delivery systems based on exosomes offer potential for enhancing the therapeutic efficacy of tumors when combined with other treatments such as chemotherapy.

One of the most promising and innovative strategies for cancer vaccine development is the use of nanotechnology to enhance the delivery of tumor antigens and adjuvants to APCs [2, 53, 54]. The use of nanoparticles as a carrier of tumor cell components has been shown to enhance the immunogenicity of tumor antigens and promote efficient antigen presentation, leading to improved antitumor immune responses.

Nanoparticles have also been used to improve the delivery of cancer vaccines in combination with other strategies. For example, the combination of nanoparticles with checkpoint inhibitors has been shown to improve antitumor immune responses. A recent study used polymeric nanoparticles loaded with ovalbumin (OVA) and a TLR7 agonist in combination with anti-PD-L1 antibodies to induce OVA-specific CD8^+^ T cell responses in mice. The results showed that the combination of nanoparticles and checkpoint inhibitors enhanced T cell activation and provided significant protection against OVA-expressing tumor cells.

Another promising approach is the combination of nanoparticles with other vaccine strategies, such as DC-based vaccines. DC-based vaccines involve the isolation and activation of DCs from a patient's own blood, which are then loaded with tumor antigens and reinfused back into the patient to induce antitumor immune responses. The use of nanoparticles as a carrier of tumor antigens in DC-based vaccines has been shown to enhance the immunogenicity of tumor antigens and promote efficient antigen presentation. For example, a recent study used polymeric nanoparticles loaded with a HER2 peptide and CpG oligodeoxynucleotides (ODNs) in combination with DC-based vaccines to induce HER2-specific immune responses in breast cancer patients [55]. The results showed that the combination of nanoparticles and DC-based vaccines was safe and effective in inducing antitumor immune responses.

Nanoparticle-based cell vaccines have been developed for cancer immunotherapy, aiming to enhance the activation of antitumor immune responses. These vaccines are typically composed of tumor cells or lysates loaded onto various types of nanoparticles, such as liposomes, polymeric nanoparticles, or inorganic nanoparticles. The combined use of nanoparticles and cell vaccines can improve vaccine efficacy by promoting antigen uptake, processing, and presentation by APCs. Several studies have demonstrated the potential of nanoparticle-based cell vaccines in preclinical and clinical settings. For example, a recent study showed that liposomal nanoparticle encapsulation of tumor cell lysate improved the uptake and presentation of tumor antigens, resulting in enhanced antitumor immunity and improved survival in a murine tumor model [56]. In another study, polymeric nanoparticles loaded with tumor cell lysate induced potent antitumor immune responses in a phase I clinical trial in patients with non-small cell lung cancer [57]. These promising results highlight the potential of nanoparticle-based cell vaccines as a promising strategy for cancer immunotherapy. Additionally, the vaccines of sipuleucel-T were approved by the United States Food and Drug Administration (FDA). It was consisted of autologous peripheral blood mononuclear cells, such as DCs, accompanied with the hybrid prostatic acid phosphatase antigen and GM-CSF. Furthermore, many other cell-based cancer vaccines have been developed for the activation of the antitumor immune respose [58].

To summarize, cancer vaccines that utilize tumor cell components and nanotechnology hold significant potential as a therapeutic strategy for the prevention and treatment of cancer. The incorporation of nanomaterials and nanocarriers has allowed for the delivery of antigens to immune cells, improved targeting of tumor cells, and enhanced the immune response to cancer cells. By combining these innovative strategies with traditional cancer treatments, such as chemotherapy and radiation therapy, the effectiveness of cancer vaccines can be further enhanced. Despite the promising results obtained from preclinical and clinical studies (Table 1), many challenges still need to be addressed to optimize the efficacy and safety of cancer vaccines. In particular, the selection of appropriate tumor antigens, the identification of optimal delivery systems, and the development of effective adjuvants are critical factors that require further investigation. Moreover, the development of standardized protocols for clinical trials and regulatory approval processes will be necessary to ensure the widespread implementation of cancer vaccines in clinical settings.

The combination of tumor cell components and nanotechnology has led to the development of innovative cancer vaccines that have shown great potential for preventing and treating cancer. The continued development of these vaccines will undoubtedly lead to even greater success in the fight against cancer. As research in this field continues to expand, further studies will be needed to optimize the design and delivery of cancer vaccines. This includes the development of more effective nanocarriers, such as liposomes, nanoparticles, and dendrimers, as well as the use of novel targeting strategies, such as aptamers and antibodies, to enhance the specificity and efficacy of cancer vaccines. In addition, further research is needed to investigate the immune response and potential side effects of these vaccines in both preclinical and clinical studies.

It is premature to claim complete victory in tumor vaccine development solely relying on nanotechnology. While current technologies have certainly facilitated advancements in the field of vaccines, there remain numerous challenges and obstacles that need to be addressed. The use of nanotechnology in cancer vaccine development also raises ethical and safety concerns [65–67], particularly with regards to the long-term effects of nanomaterials in the body, such as PLGA, liposomes, and viral structural proteins. While studies have shown that many nanocarriers are biocompatible and biodegradable, more research is needed to fully understand the long-term safety implications of these materials. Many vaccines have achieved satisfactory results in animal experiments but have failed in clinical trials. The reason may lie in the differences between the immune systems of experimental animals and humans. The main reason for the lack of reproducibility in clinical treatment is the absence of animal models that accurately reflect the pathological characteristics of patients. Although there are humanized animal models available, research is still limited, and the development of more precise animal models will help validate the efficacy of the developed vaccines. In addition, the identification of tumor-specific antigen sequences was significantly for the development of tumor vaccines, while lacking adequate technical support. Meanwhile, large-scale production and precise quality control in commercialized vaccines also limit the development and clinical application of cancer vaccines [68]. Therefore, interdisciplinary collaboration involving artificial intelligence, big data analysis, and other fields is an important way to promote the development of cancer vaccines in the new era.

The development of cancer vaccines that utilize tumor cell components and nanotechnology represents a promising new approach to the prevention and treatment of cancer. By incorporating innovative strategies such as nanocarriers, targeting ligands, and adjuvants, these vaccines hold great potential for enhancing the immune response to cancer cells and improving patient outcomes. While there are still many challenges to be overcome, continued research in this field will undoubtedly lead to even greater success in the fight against cancer.

The clinical translation of cancer vaccines based on tumor cell components and nanotechnology will require the development of standardized protocols for clinical trials and regulatory approval processes. The FDA has already approved a number of cancer vaccines for clinical use, including sipuleucel-T, which is used to treat advanced prostate cancer, and T-VEC, which is used to treat melanoma. However, more research is needed to fully understand the mechanisms of action and clinical efficacy of these vaccines.

In addition, the development of cancer vaccines will require collaboration between researchers, clinicians, and industry partners to overcome technical and logistical challenges. This includes the development of cost-effective manufacturing processes for these vaccines, as well as the establishment of large-scale clinical trials to evaluate their efficacy in diverse patient populations.

As the field of cancer vaccine research continues to advance, the potential for these therapies to transform cancer treatment and prevention remains high. By utilizing the unique properties of nanomaterials and tumor cell components, researchers are developing innovative approaches to cancer immunotherapy that could 1 day lead to a cure for cancer.

In conclusion, the development of nanoparticle-based cancer vaccines has shown promising results in preclinical and clinical studies. Nanoparticles as vaccine carriers have been reported to enhance the immunogenicity of tumor antigens and promote a potent immune response against cancer cells. Additionally, nanoparticle-based vaccines have the potential to enable personalized and combination immunotherapies due to their versatility in targeted delivery and codelivery of multiple antigens or adjuvants. However, there are still challenges to be addressed in terms of safety, scalability, and clinical translation of these vaccines. Future research should focus on optimizing vaccine formulation and manufacturing processes, as well as evaluating the long-term safety and efficacy of nanoparticle-based cancer vaccines in clinical trials. With continued advancements and interdisciplinary collaborations, nanoparticle-based approaches hold great promise in revolutionizing cancer treatment and improving patient outcomes.

## Figures and Tables

**Table 1:** Some pivotal trials for tumor vaccines.

Vaccine platform type	Product/compound name	Antigen(s)	Identifier (phase, name)	Findings	References
Tumor cell-based	Belagenpumatucel-L (Lucanix™)	/	NCT00676507 (phase 3)	Second interim analysis, futility	[59]

Tumor cell-based	Canvaxin™ (CancerVax)	/	NCT00052130 (phase 3, MMAIT-III)	Study was terminated (low probability)	[60, 61]

DC-based	Peptide-loaded DC vaccine	Several MHC restricted peptides	(phase 3)	Study was prematurely closed	[62]

Protein	BEC2	GD3	NCT00037713 (phase 3, SILVA)	Population heterogeneity and study design	[63]

Protein	THERATOPE®	STn	NCT00003638 (phase 3)	Tumor burden; treatment duration	[64]

## Data Availability

The data that support the findings of this study are available on request from the corresponding author. The data are not publicly available due to privacy or ethical restrictions.
